# Supplementary material for: Engineering of an enhanced synthetic Notch receptor by reducing ligand-independent activation
Source: Commun Biol. 2020 Mar 13;3:116. doi: 10.1038/s42003-020-0848-x (PMC7069970; doi:10.1038/s42003-020-0848-x)
Supplement: Supplementary file 1 — Supplementary Information [file 42003_2020_848_MOESM1_ESM.pdf]

Engineering of an enhanced synthetic Notch receptor by reducing ligand-independent activation

Supplementary Figures

Supplementary Figure 1. Gating for flow cytometry data quantification

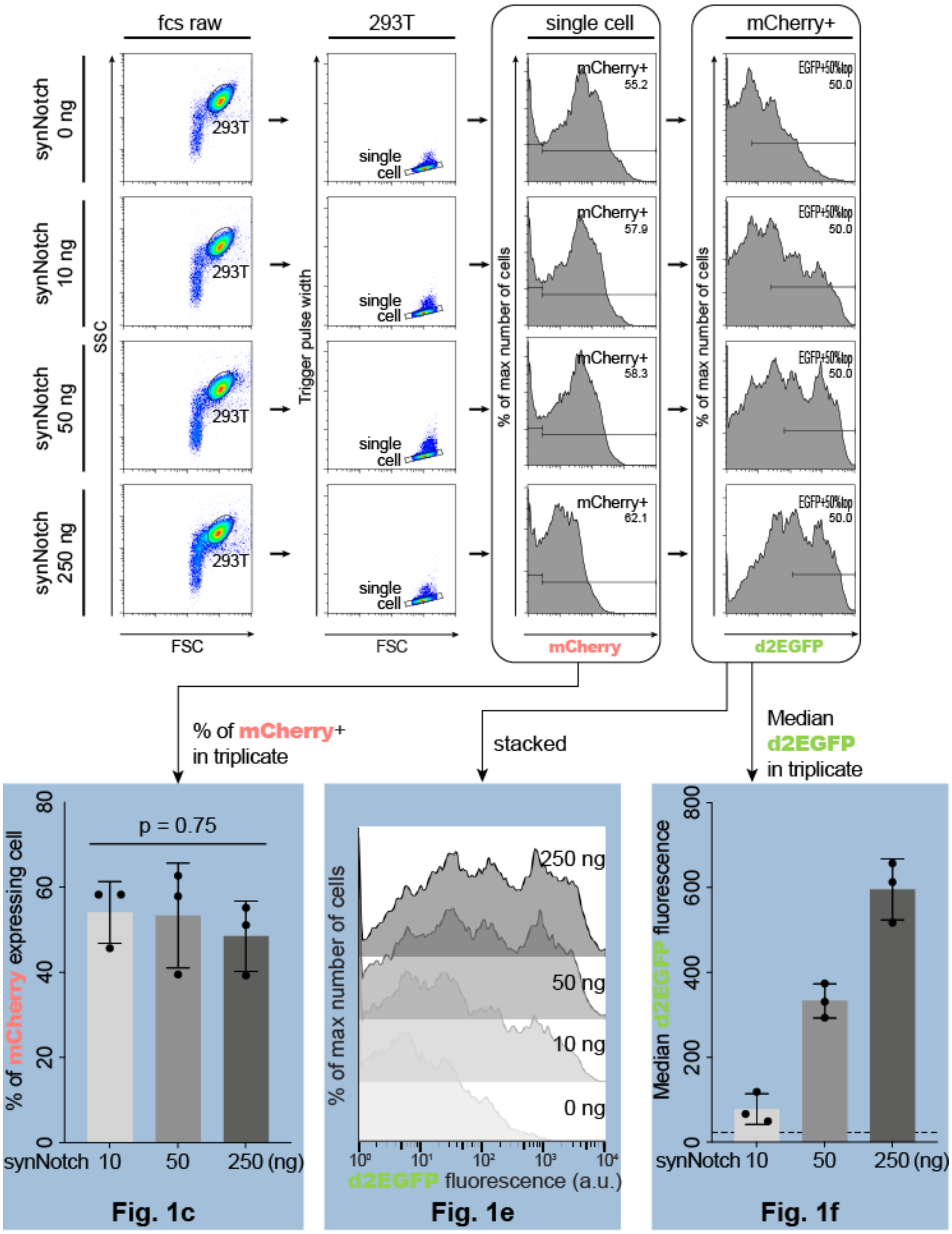

We transiently co-transfected cells with plasmid DNAs to reproduce the ligand-independent activation (LIA). For experiments in Fig. 1c-f, cells were analyzed 48 hr after co-transfection by flow cytometry. We used signals from forward scatter light (FSC) and side scatter light (SSC) to eliminate cell debris by “293T” gate. We used

signals from FSC and measured trigger pulse width to collect singlets by “single cell” gate. Because all synNotch expressing cells were positive for red fluorescence, we collected them by “mCherry+” gate. We recorded the percentages of mCherry positive cells in the parent group as an evaluation of transfection efficiency. The experiment was independently performed in triplicate, and the data was presented as a scatter dot plot, with bar height representing the mean and error bars as standard deviations (Fig. 1c). Then we measured the green fluorescence from those mCherry positive cells. When different amounts of PGK driven synNotch were transfected, the expression histograms of d2EGFP were different. We stacked those histograms into Fig. 1e. To quantify the results from triplicated experiments, we used a previously described method (Gao Y *et al*, 2016): within the EGFP positive cells, the median fluorescence intensity of the top 50% cells was used to represent the entire distribution. Data was presented as a scatter dot plot (bar: mean ± SD). For Fig. 1f, d2EGFP fluorescence was due to LIA, because antigen-expressing sender was not presented.

Supplementary Figure 2. 293T cells express various antigens on the membrane as sender cells

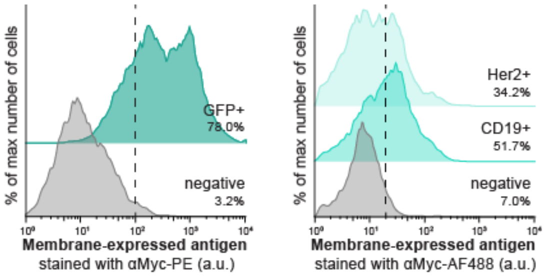

We inserted a Myc tag between the signal peptide and the antigen (GFP, Her2, or CD19) to facilitate the detection of the antigen expressing on the cell membrane. Cells were transiently transfected for 48 hr, fixed with 4% PFA in PBS, and stained using αMyc antibodies without permeabilization. Stacked histograms show the fluorescence signal from the extracelluar Myc tag. The numbers on the lower right corners of each histogram are the percentages of Myc-tag positive cells or background signals due to antibody staining.

Supplementary Figure 3. Extended co-culture of cells cannot enhance the antigen-induced activation

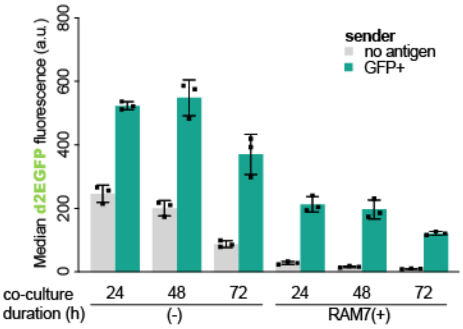

Similar to the experiments as in Fig. 3b, synNotch cells were co-cultured with sender cells with or without GFP antigen for various durations. After co-culture of sender cells and synNotch cells for 24, 48, and 72 hours, the populations were fixed with 4% PFA in PBS. The samples from 24 and 48 hours were kept in the dark at 4 °C until the samples from 72 hours were ready. In groups marked by (-), cells were expressing synNotch without the RAM7 sequence. The median d2EGFP fluorescence intensity from synNotch cells was calculated and presented as a scatter dot plot, with bar height as mean and error bars as standard deviations.

Supplementary Table

Supplementary Table 1. synNotch constructs used in this study

Abbreviation: mN1c, mouse Notch 1 core. hN1c, human Notch 1 core. TMD, transmembrane domain (equals to Notch core without NRR). NRR, negative regulatory region.

First appeared	Name	Extracellular domain	Intracellular domain	Notch core + RAM	RAM sequence
Fig. 1d	LaG16-mN1c-tTAA	LaG16	tTAA	mN1c	–
Fig. 1k	LaG16-EGF-mN1c-tTAA	LaG16-EGF	tTAA	mN1c	–
Fig. 2a	LaG16-mN1c[S1]-tTAA	LaG16	tTAA	mN1c S1 mutant (RQRR| → AAAA)	–
Fig. 2a	LaG16-mN1c[S2]-tTAA	LaG16	tTAA	mN1c S2 mutant (A|V → ED)	–
Fig. 2a	LaG16-mN1c[S3]-tTAA	LaG16	tTAA	mN1c S3 mutant (GCG|V → LLFF)	–
Fig. 2c	LaG16-mN1c-hN1RAM8-tTAA	LaG16	tTAA	mN1c-hN1RAM8	`QHGQLWFP`
Fig. 2e	LaG16-mN1c-hN1RAM7-tTAA	LaG16	tTAA	mN1c-hN1RAM7	`QHGQLWF`
Fig. 2e	LaG16-mN1c-hN1RAM6-tTAA	LaG16	tTAA	mN1c-hN1RAM6	`QHGQLW`
Fig. 2e	LaG16-mN1c-hN1RAM5-tTAA	LaG16	tTAA	mN1c-hN1RAM5	`QHGQL`
Fig. 2e	LaG16-mN1c-hN1RAM4-tTAA	LaG16	tTAA	mN1c-hN1RAM4	`QHGQ`
Fig. 2e	LaG16-mN1c-hN1RAM3-tTAA	LaG16	tTAA	mN1c-hN1RAM3	`QHG`
Fig. 2e	LaG16-mN1c-hN1RAM2-tTAA	LaG16	tTAA	mN1c-hN1RAM2	`QH`
Fig. 2e	LaG16-mN1c-hN1RAM1-tTAA	LaG16	tTAA	mN1c-hN1RAM1	`Q`
Fig. 2g	LaG16-hN1c-hN2RAM7-tTAA	LaG16	tTAA	hN1c-hN2RAM7	`KHGSLWL`
Fig. 2g	LaG16-hN1c-mN2RAM7-tTAA	LaG16	tTAA	hN1c-mN2RAM7	`KHGFLWL`
Fig. 2g	LaG16-hN1c-hN3RAM7-tTAA	LaG16	tTAA	hN1c-hN3RAM7	`EHSTLWF`
Fig. 2g	LaG16-hN1c-hN4RAM7-tTAA	LaG16	tTAA	hN1c-hN4RAM7	`EHGALWL`
Fig. 2h	LaG16-hN1c-hN1RAM7[FW]-tTAA	LaG16	tTAA	hN1c-hN1RAM7[FW]	`QHGQLFW`
Fig. 2h	LaG16-hN1c-hN1RAM7[WW]-tTAA	LaG16	tTAA	hN1c-hN1RAM7[WW]	`QHGQLWW`

First appeared	Name	Extracellular domain	Intracellular domain	Notch core + RAM	RAM sequence
Fig. 2h	LaG16-hN1c-hN1RAM7[FF]-tTAA	LaG16	tTAA	hN1c-hN1RAM7[FF]	QHGQLFF
Fig. 2h	LaG16-hN1c-hN1RAM7[LL]-tTAA	LaG16	tTAA	hN1c-hN1RAM7[LL]	QHGQLLL
Fig. 2h	LaG16-hN1c-hN1RAM7[AA]-tTAA	LaG16	tTAA	hN1c-hN1RAM7[AA]	QHGQLAA
Fig. 2h	LaG16-hN1c-hN1RAM7[GG]-tTAA	LaG16	tTAA	hN1c-hN1RAM7[GG]	QHGQLGG
Fig. 2h	LaG16-hN1c-hN1RAM7[PP]-tTAA	LaG16	tTAA	hN1c-hN1RAM7[PP]	QHGQLPP
Fig. 2h	LaG16-hN1c-hN1RAM7[SS]-tTAA	LaG16	tTAA	hN1c-hN1RAM7[SS]	QHGQLSS
Fig. 2h	LaG16-hN1c-hN1RAM7[EE]-tTAA	LaG16	tTAA	hN1c-hN1RAM7[EE]	QHGQLEE
Fig. 2i	hN1c-hN1RAM7-tTAA	-	tTAA	hN1cNRR-hN1cTMD-hN1RAM7	QHGQLWF
Fig. 2i	hN1c-tTAA	-	tTAA	hN1cNRR-hN1cTMD	–
Fig. 2i	hN1TMD-hN1RAM7-tTAA	-	tTAA	hN1cTMD-hN1RAM7	QHGQLWF
Fig. 2i	hN1TMD-tTAA	-	tTAA	hN1cTMD	–
Fig. 3b	LaG16-hN1c-hN1RAM7-tTAA	LaG16	tTAA	hN1c-hN1RAM7	QHGQLWF
Fig. 3e	LaG16-hN1c-hN1RAM7-CymR-VP64	LaG16	CV2	hN1c-hN1RAM7	QHGQLWF
Fig. 3h	αCD19-hN1c-hN1RAM7-tTAA	αCD19	tTAA	hN1c-hN1RAM7	QHGQLWF
Fig. 3k	αHer2-hN1c-hN1RAM7-tTAA	αHer2	tTAA	hN1c-hN1RAM7	QHGQLWF
Fig. 3c	LaG16-hN1c-tTAA	LaG16	tTAA	hN1c	–
Fig. 3f	LaG16-hN1c-CymR-VP64	LaG16	CV2	hN1c	–
Fig. 3i	αCD19-hN1c-tTAA	αCD19	tTAA	hN1c	–
Fig. 3l	αHer2-hN1c-tTAA	αHer2	tTAA	hN1c	–

Supplemental Reference

Gao, Y, Xiong, X, Wong, S, Charles, EJ, Lim, WA, and Qi, LS. Complex transcriptional modulation with orthogonal and inducible dCas9 regulators [J]. *Nature Methods*, 2016, 13(12): 1043.
