# Supplementary material for: Engineering of an enhanced synthetic Notch receptor by reducing ligand-independent activation
Source: Commun Biol. 2020 Mar 13;3:116. doi: 10.1038/s42003-020-0848-x (PMC7069970; doi:10.1038/s42003-020-0848-x)
Supplement: Supplementary file 2 — Reporting Summary [file 42003_2020_848_MOESM2_ESM.pdf]

nature research

Corresponding author(s): Liang Cai

Last updated by author(s): Jan 18, 2020

Reporting Summary

Nature Research wishes to improve the reproducibility of the work that we publish. This form provides structure for consistency and transparency in reporting. For further information on Nature Research policies, see Authors & Referees and the Editorial Policy Checklist.

Statistics

For all statistical analyses, confirm that the following items are present in the figure legend, table legend, main text, or Methods section.

n/a	Confirmed	
☐	☒	The exact sample size (*n*) for each experimental group/condition, given as a discrete number and unit of measurement
☐	☒	A statement on whether measurements were taken from distinct samples or whether the same sample was measured repeatedly
☐	☒	The statistical test(s) used AND whether they are one- or two-sided<br>*Only common tests should be described solely by name; describe more complex techniques in the Methods section.*
☒	☐	A description of all covariates tested
☐	☒	A description of any assumptions or corrections, such as tests of normality and adjustment for multiple comparisons
☐	☒	A full description of the statistical parameters including central tendency (e.g. means) or other basic estimates (e.g. regression coefficient) AND variation (e.g. standard deviation) or associated estimates of uncertainty (e.g. confidence intervals)
☐	☒	For null hypothesis testing, the test statistic (e.g. *F*, *t*, *r*) with confidence intervals, effect sizes, degrees of freedom and *P* value noted<br>*Give P values as exact values whenever suitable.*
☒	☐	For Bayesian analysis, information on the choice of priors and Markov chain Monte Carlo settings
☒	☐	For hierarchical and complex designs, identification of the appropriate level for tests and full reporting of outcomes
☒	☐	Estimates of effect sizes (e.g. Cohen's *d*, Pearson's *r*), indicating how they were calculated

Our web collection on statistics for biologists contains articles on many of the points above.

Software and code

Policy information about availability of computer code

Data collection	BD FACS software for collecting flow cytometry data.
Data analysis	Raw flow cytometry data was analyzed using FlowJo (version 10). The data was further analyzed using Excel for Mac (version 15.41) and GraphPad Prism (version 7).

For manuscripts utilizing custom algorithms or software that are central to the research but not yet described in published literature, software must be made available to editors/reviewers. We strongly encourage code deposition in a community repository (e.g. GitHub). See the Nature Research guidelines for submitting code & software for further information.

Data

Policy information about availability of data

All manuscripts must include a data availability statement. This statement should provide the following information, where applicable:

- Accession codes, unique identifiers, or web links for publicly available datasets
- A list of figures that have associated raw data
- A description of any restrictions on data availability

Data supporting the findings of this study are available in the paper and its supplementary information files. The raw data are deposited at Mendeley.

Field-specific reporting

Please select the one below that is the best fit for your research. If you are not sure, read the appropriate sections before making your selection.

☒ Life sciences ☐ Behavioural & social sciences ☐ Ecological, evolutionary & environmental sciences

Life sciences study design

All studies must disclose on these points even when the disclosure is negative.

Sample size	Three independent replicates were performed for all experiments.
Data exclusions	No data were excluded from the datasets.
Replication	All experiments were independently performed in triplicate with a comparable outcome.
Randomization	The positions of samples on multi-well plates were different between experiments to minimize the systematic errors.
Blinding	No blinding.

Reporting for specific materials, systems and methods

We require information from authors about some types of materials, experimental systems and methods used in many studies. Here, indicate whether each material, system or method listed is relevant to your study. If you are not sure if a list item applies to your research, read the appropriate section before selecting a response.

Materials & experimental systems

n/a	Involved in the study	
☐	☒	Antibodies
☐	☒	Eukaryotic cell lines
☒	☐	Palaeontology
☒	☐	Animals and other organisms
☒	☐	Human research participants
☒	☐	Clinical data

Methods

n/a	Involved in the study	
☒	☐	ChIP-seq
☐	☒	Flow cytometry
☒	☐	MRI-based neuroimaging

Antibodies

Antibodies used	Antibodies used for flow cytometry: αMyc AF488 (Cell Signaling Technology #2279; diluted 1:100), αMyc PE (Cell Signaling Technology #3739; diluted 1:100) or αMyc AF647 (Cell Signaling Technology #2233; diluted 1:100).
Validation	These antibodies are used on a weekly basis in our lab. Additional information of the antibodies are available on the manufacture's websites.

Eukaryotic cell lines

Policy information about cell lines

Cell line source(s)	We used the HEK293T and K562 cell lines (ATCC).
Authentication	These cell lines were not additionally authenticated.
Mycoplasma contamination	The lab regularly tests all cell lines in use for mycoplasma infection. No infection was detected during experiments presented in this study.
Commonly misidentified lines (See ICLAC register)	NA

Flow Cytometry

Plots

Confirm that:

☒ The axis labels state the marker and fluorochrome used (e.g. CD4-FITC).

☒ The axis scales are clearly visible. Include numbers along axes only for bottom left plot of group (a 'group' is an analysis of identical markers).

☐ All plots are contour plots with outliers or pseudocolor plots.

☒ A numerical value for number of cells or percentage (with statistics) is provided.

Methodology

Sample preparation	Fluorescent cells were dissociated using 0.05% Trypsin-EDTA, fixed using 4% PFA in PBS, washed by PBS, and stored at 4 °C in the dark prior to analysis. To detect surface-expressed synNotch, cells were resuspend using PBS, fixed and stained with antibodies.
Instrument	Flow cytometry analysis was performed using a BD FACSJazz flow cytometer. Fluorescence from d2EGFP or AF488 was measured by 488 nm excitation laser, mCherry or PE was measured by 561 nm laser and AF647 was measured by 640 nm laser.
Software	The data was collected using the BD FACS software and analyzed using the FlowJo software.
Cell population abundance	At least 20,000 events were measured for each set of data, and at least 50,000 events were analyzed when synNotch cells were co-cultured with sender cells. The populations were gated for mCherry-positive cells. The percentage of mCherry-positive was dependent on the transfection efficiency in each experiment, usually between 40-60%.
Gating strategy	We used the forward scatter (FSC) and side scatter (SSC) signals to remove debris, FSC and trigger pulse width signals to gate out singlets. For the transient transfection experiments, cells were gated for mCherry-positive cells to ensure that only cells that were successfully transfected with synNotch were used for further analysis.

☒ Tick this box to confirm that a figure exemplifying the gating strategy is provided in the Supplementary Information.
